# Comparison of Giant Magnetoimpedance and Anisotropic Magnetoresistance Sensors for Residual Stress Distribution Determination in Magnetic Steels

**DOI:** 10.3390/s26010032

**Published:** 2025-12-20

**Authors:** Sergey Gudoshnikov, Tatiana Damatopoulou, Evangelos Hristoforou

**Affiliations:** 1 Pushkov Institute of Terrestrial Magnetism, Ionosphere and Radio Wave Propagation, IZMIRAN, Kaluzhskoe, sh. 4, Troitsk, 108840 Moscow, Russia; gudosh@izmiran.ru; 2 Laboratory of Electronic Sensors, National TU of Athens, Zografou Campus, 15780 Athens, Greece; damatat5@yahoo.gr; 3 National Key Laboratory of Spintronics, Hangzhou International Innovation Institute, Beihang University, Hangzhou 311115, China

**Keywords:** residual stress, steel stress coupons, magnetic stress calibration (MASC) curves, giant magnetoimpedance (GMI), anisotropic magnetoresistance (AMR)

## Abstract

Our team has initiated work to determine residual stresses by means of monitoring magnetic properties, namely differential permeability, magnetoacoustic emission, and surface field components. Concerning surface field measurements, Hall, AMR, and TMR sensors have been used, with AMR and TMR sensors enabling 3D field determination. In this paper, we compare the surface magnetic field components with residual stresses in 2 mm thick AISI 4130 steel coupons. The steel samples were in a dog-bone structure with residual stresses induced by localized RF induction heating to create a temperature gradient, followed by quenching to transform the temperature gradient into a residual stress one. GMI and AMR sensors were used to determine the localized magnetic field component distribution on the surface of the steel coupons and at the same areas where the residual stresses were determined. The GMI sensor was able to monitor the field component perpendicular to the surface of the steel coupon, while the AMR sensor was able to monitor the three field components at the same points. The results illustrated that both sensors were able to monitor residual stresses, with the GMI sensor illustrating better sensitivity at a higher cost, while the AMR sensor had a lower sensitivity with a significantly lower cost as an integrated sensor.

## 1. Introduction

Our team initiated work on magnetomechanical and magnetostriction effects in 1987, with our first results being disseminated in 1990. Since then, we have focused on the application of the magnetomechanical effect and magnetostriction in terms of monitoring residual stresses in steel, developing the so-called magnetic stress calibration (MASC) curves. An interesting result is the Universality Law governing the dependence of MASC curves on residual stresses. This is observed after normalization of the stress or strain axis (*X*-axis) by the corresponding yield point of the related steel grade and their magnetic property axis (*Y*-axis) by the maximum recorded amplitude of that property. This enables the determination of the MASC curve for an unknown steel through just a stress–strain measurement with simultaneous monitoring of a magnetic property. Concerning the particular type of magnetic property, we have measured several different types of properties and corresponding devices, such as induction response concerning reference single sheet testers, electromagnetic yokes and Barkhausen probes, magnetoelastic emission devices, as well as field sensors at the surface of the steel under test.

The field measurement on the surface of the steel under test gives rise to an interesting methodology, named the magnetic memory method (MMM), developed by Dubov [1,2], Dobmann [3,4], and others [5]. The method has also been standardized, allowing its use in the industry [6]. The basic principle of the MMM technique is the change in magnetization and differential permeability of the material due to stresses [7]. Using the MMM, there is no need for additional magnetization to be applied to the steel except for Earth’s or ambient field. This method appears to be attractive for a few reasons. Firstly, the sensitive field magnetometers that are built with an integrated circuit offer the possibility of inspecting magnetic properties inside the grains of the steels due to their miniaturized size, without the need for a biasing field, when their equivalent magnetic noise is better than 50 nTHz^−1/2^ @1Hz and their dynamic range is well above 1 mT. For this reason, we have used AMR- and TMR-integrated sensors available in the market to perform this type of measurement [8]. The three-dimensional AMR sensors, for example, offer a magnetic noise better than 50 nTHz^−1/2^ @1 Hz with a dynamic range in the order of ±10 mT, making it attractive for these applications at considerably low cost.

However, the giant magnetoimpedance (GMI) technology [9,10,11], using glass-covered amorphous wires, is also highly promising, offering optimum equivalent magnetic noise down to pTHz^−1/2^ @1 Hz levels; however, this comes at a higher cost since it is not an integrated device [12,13]. The sensing area is approximately equal to the inner magnetic core of the glass-covered wire, which is in the order of 5–15 μm; that is, somehow comparable to the sensing area of AMR and TMR sensors [14]. For this reason, we decided to compare GMI sensors with AMR sensors to examine their applicability in different applications, namely in testing stress coupons, which are steel parallelepipeds with residual stresses on their surface caused by the transformation of temperature gradient to stress gradient. This work is presented in the next chapters, starting with the new experimental results and then continuing with a discussion of their applicability.

## 2. Experimental Setup

**Figure 1:**
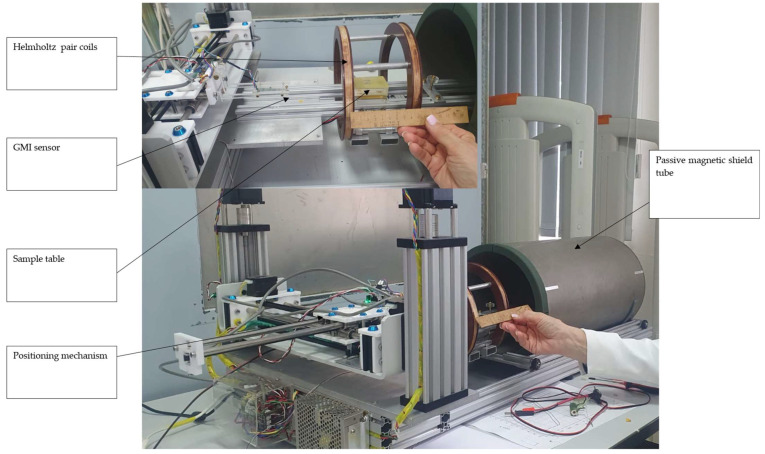
The shielded measuring setup in Moscow. The passive compensation tube can be seen in the main photo, while the active compensation unidirectional Helmholtz pair coils can be seen in the inset.

**Figure 2:**
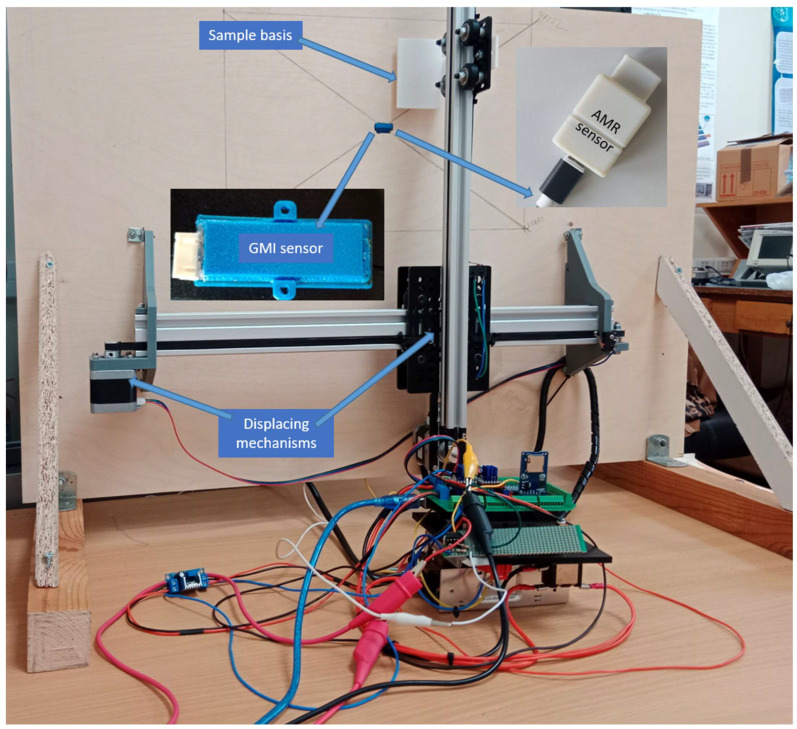
The non-shielded measuring setup in Athens, allowing field measurement simulation. The GMI and AMR sensors can be interchangeable at their position in the wooden support of the experiment.

Experiments were initiated in Moscow and then continued in Athens, with the corresponding experimental setups illustrated in Figure 1 and Figure 2, respectively. In the setup shown in Figure 1, the passive field compensation tube (magnetic μ-metal shield) can be seen in the main photo, with the magnetizing unidirectional Helmholtz pair in the inset. In these experiments, the GMI sensor was moving on top of the steel under test along the X–Y axes. The GMI sensor is based on a glass-covered Co-Fe-Cr-Si-B microwire. The probe itself is a 5 mm long microwire with a miniature copper pickup coil less than 1 mm long wound at one end of the microwire. During measurements, the microwire is excited by a sinusoidal current with an amplitude of 1 mA and frequency of 4 MHz, while the output signal is measured from the pickup coil, i.e., the output signal is proportional to the real part of the off-diagonal component of the magnetic impedance tensor of the microwire. A detailed description of the GMI magnetometer is given in [15].

The experimental setup in Athens is illustrated in Figure 2. In this arrangement, two motors were moving an aluminum head, allowing for two-dimensional displacement, carrying the steel under test. The setup allowed for the interchange of the two types of sensors, which had very similar packaging dimensions. The setup in Athens was not shielded either passively or actively because these are the conditions of measuring in the field. The AMR sensor was an ST Microelectronics LSM 303D device. Its packaging had dimensions of 23 mm × 12 mm × 41 mm, and it featured a maximum power consumption of 3.3 V × 43 mA = 142 mW. The sensor had a temperature coefficient of 100 ppm/K up to 40 μT. Its electronics included a sensing element and a microcontroller, with the capability of operating in either wired mode or wirelessly using WiFi/Bluetooth transmission.

The experiments were conducted on steel coupon samples. These initially were 30 cm long, 5 cm wide, and 2 mm thick low-carbon steel parallelepipeds that hosted various residual stresses up to the yield point. They were prepared by heating one end or the middle of a steel plate from 400 °C up to 450 °C by Joule and by RF induction heating, allowing for a slow temperature gradient along the length of the steel plate due to the low thermal conductivity of steel. For Joule heating, the steel samples were heated at their end for practical handling reasons, while for the RF induction heating, the samples were heated in their middle to take advantage of the whole area of the RF induction heating. As soon as the temperature of the other side was slightly elevated, i.e., 5–10 °C, the steel parallelepiped was inserted in iced water, thus transferring the temperature gradient to a residual stress gradient. These steel coupons serve to measure the surface and bulk residual stress distribution using X-ray diffraction in the Bragg–Brentano setup and neutron diffraction for surface (2D) and bulk (3D) residual stress measurements, respectively. In this paper, we will focus only on the magnetic testing of these steel coupons, made from different steel grades, such as oriented electric steel, mild steel, and AISI 4130 steel. It should be noted that in the tested steel coupons, heating was performed in the middle of the samples for practical reasons, without loss of generality.

## 3. Experimental Results

**Figure 3:**
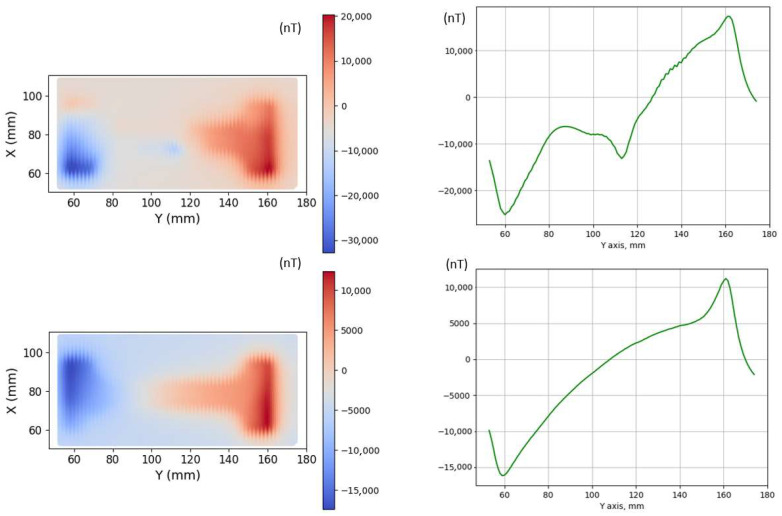
Vertical magnetic field component measured using the GMI sensor with a lift-off of 2 mm over the dog-bone coupon No. 1 before demagnetization (**top**) and after mild demagnetization (**bottom**). The graphs on the right illustrate the response of single-line mapping (X = 80 mm), showing that the remnant magnetic field in the initial state is much higher than the field after demagnetization of the sample.

Figure 3 illustrates the magnetic field scanning of the surface of a mild steel dog-bone coupon (No. 1) using the GMI sensor. The scanning took place inside the shielded tube with a GMI sensor lift-off of 2 mm. The process included 72 measurements at each line, with 20 lines scanned altogether by lateral movement of the GMI sensor. The figure shows the vertical magnetic field component after heating at 450 °C and quenching in iced water (top). It is evident that the magnetic field distribution in the left and middle parts of the specimen is non-uniform, which is due to the presence of residual stress gradients in the specimen. The response below refers to the same material after partial demagnetization. It can be seen that the behavior of the magnetic field was more informative for the non-demagnetized sample. This indicates that measuring the initial state in an ambient magnetic environment is favorable, as it allows for the observation of changes in remnant magnetization. Similar measurements but with less sensitivity were observed using the AMR sensor.

**Figure 4:**
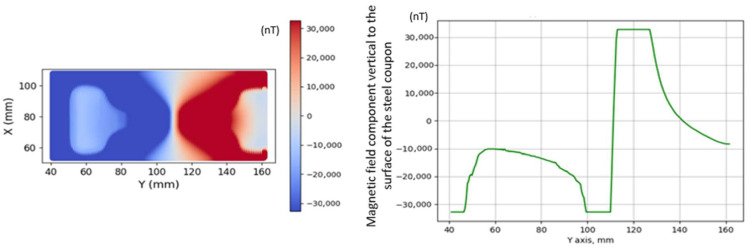
Response of the GMI sensor under a biasing field of 0.6 mT along the axis of the dog-bone steel coupon No. 1; the GMI sensor lift-off is 6.3 mm. The right-side graph illustrates the response of a single-line mapping (X = 80 mm), depicting that the response of the stress field region is not captured well due to the saturation of the GMI sensor.

An interesting result is illustrated in Figure 4 concerning the dependence of the GMI response on the additional magnetization field for the same dog-bone steel coupon No. 1. The additional magnetic field in the order of 0.6 mT is along the axis of the dog-bone steel coupon. In the field maps, the GMI sensor lift-off equals 6.3 mm. This response clearly demonstrates the inability of the GMI sensor to take measurements in magnetically harsh environments due to its limited dynamic range of ± 33 µT. On the contrary, the AMR sensor, having an equivalent magnetic noise level of 50 nTHz^−1/2^ @ 1 Hz and a dynamic range ± 10 mT, was able to measure the response, as shown in Figure 3, with a sensitivity reduced to about 20% under the same conditions of lift-off and biasing fields.

**Figure 5:**
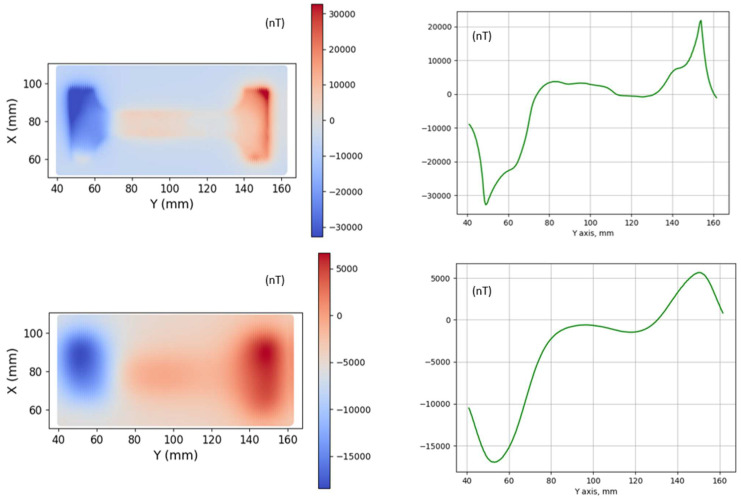
GMI sensor response of the dog-bone steel coupon No. 2 for distances of 2 mm (**top**) and 8 mm (**bottom**). The response clearly shows that the field gradient details in the short lift-off are clearer than in the long lift-off.

Figure 5 shows the magnetic response of the dog-bone steel coupon, No. 2, for distances of 2 mm (top) and 8 mm (bottom) without an extra magnetic field. The response demonstrates that the magnetic field gradient details in the short lift-off are clearer than in the long lift-off. However, the AMR sensor was able to properly measure at lift-offs below 1 mm.

**Figure 6:**
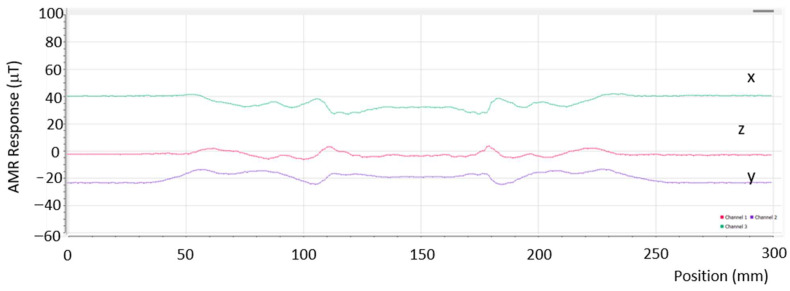
The AMR response on the oriented electric steel coupon No. 3 under no external field at a lift-off distance of 1 mm. The x, y, and z curves represent the Hx, Hy, and Hz field components on top of the point of measurement. The same response in the Z axis with the GMI sensor was achieved at 2.5 mm lift-off.

The response of the oriented electric steel laminar steel coupon, No. 3, after a heat treatment at 300 °C and subsequent quenching is shown in Figure 6. The results in the figure are depicted with the AMR sensor at a lift-off distance of 1 mm and in the absence of an ambient field. This steel coupon underwent RF induction heating using a circular RF induction coil, which induced stresses in two areas on its surface. The response of the GMI sensor for the same steel coupon using the GMI sensor was similar for a lift-off distance of 2.5 mm. It should be noted that when the response of one of the two sensors is shown, the response of the other one is not illustrated for clarity. The GMI sensor captures only the *Z*-axis component, whereas the AMR sensor provides a response in the three axes, demonstrating significant information concerning the contradictory orientations of the field gradient in the three axes.

**Figure 7:**
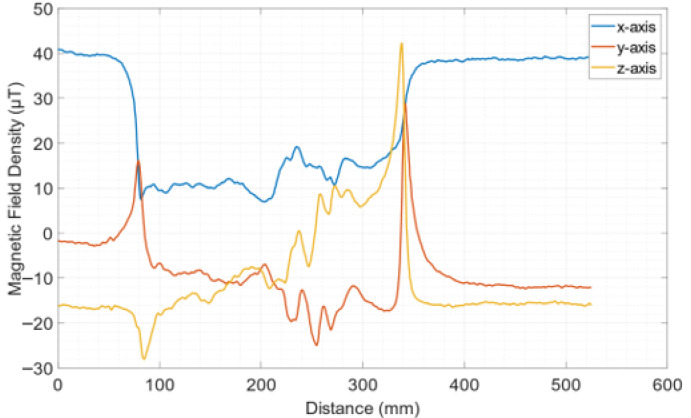
The AMR sensor response for oriented electric steel coupon No. 4, locally heated at 450 °C and then ice-water quenched. The mapping was performed at a lift-off distance of 1 mm, while the same response was achieved at lift-off 2.5 mm using the GMI sensor.

Figure 7 illustrates the AMR sensor response of another oriented electric steel coupon, No. 4, with localized temperature fixed at 450 °C by Joule heating and subsequent quenching in iced water. This response illustrates a relatively anomalous magnetic field distribution effect, which is attributed to the large localized temperature, causing micro-deformation of the steel coupon, which is 0.25 mm thin and therefore sensitive to geometrical deformation at elevated temperatures and subsequent quenching. The lift-off distance was 1 mm. The same response was also observed using the GMI sensor at a lift-off of 2.5 mm. No extra magnetization field was used in this measurement. The large peaks in the response correspond to the ends of the steel coupon.

## 4. Discussion

A comparison between the two sensors demonstrated some interesting results, allowing for the proper selection of each sensor type for its corresponding applications. The GMI sensor is capable of measuring with a sensitivity well below 1 nTHz^−1/2^ @ 1 Hz, with a rather restricted dynamic range (span), which is limited to below 0.1 mT. In fact, the sensor we used has a sensitivity in the order of 10 pTHz^−1/2^ @ 1 Hz, allowing for sensitive measurements in laboratory conditions. On the other hand, the AMR sensor we used has a sensitivity in the order of 50 nTHz^−1/2^ @ 1 Hz and a dynamic range of ±10 mT, which allows for field applications. Their temperature coefficients are comparable, allowing for proper operation in the range from −40 °C up to at least 80 °C. The comparison between these two sensors is illustrated in Table 1.

Without any loss of generality, the possible applications for these two sensors will be considered with respect to the steel manufacturing industry, especially steel pipeline production, using ferrous steel grades in the form of plates or steel coils as the pipeline raw material.

**Figure 8:**
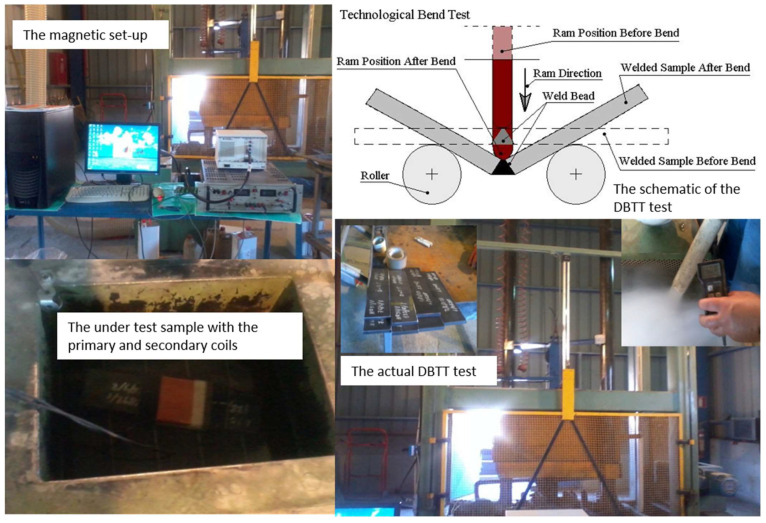
A magnetic method for determining DBTT at a pipeline manufacturer. The classic drop-off measurement method is shown on the right—schematically (**top**) and in practice (**bottom**). The corresponding insets show standard samples and part of the cooling process. On the left are magnetic devices (**top**) with primary–secondary coils (**bottom**). All these magnetic measurements, which replace the drop-off measurement method, can be significantly simplified by simply mounting the GMI sensor on top of the sample.

**Figure 9:**
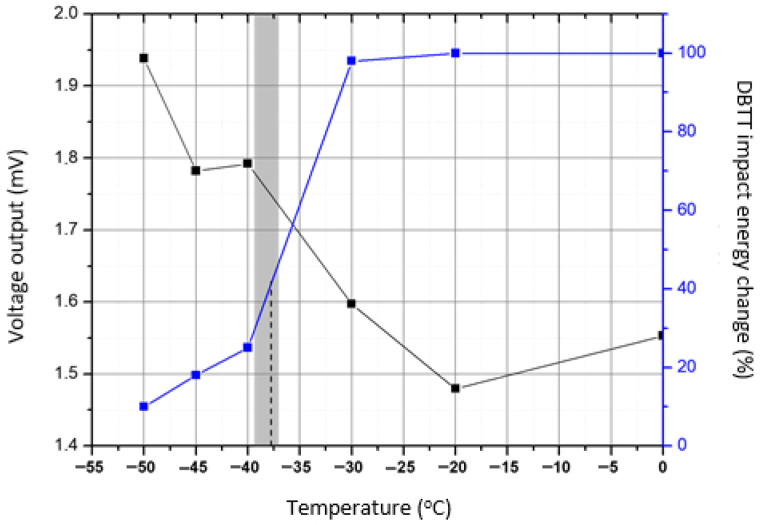
Correlation between magnetic permeability (black letters—Vout) and the classic DBTT (blue line = SA%) test at a pipeline manufacturer. The shade illustrated in the figure corresponds to the ductile to brittle temperature transition zone.

GMI sensors should be used in laboratory conditions. In fact, a certain application can be the determination of the ductile-to-brittle transition temperature (DBTT) in steel manufacturing lines. The classic and standard technique for this purpose is the Charpy impact test [17], which is used to determine the energy required to break a sample with respect to the temperature of the material. This helps to determine its ductile or brittle nature by locating the temperature region in which the ductile-to-brittle transformation occurs. In the classic DBTT method, at given discrete temperatures, the steel sample moves out of the cryo-bath and is broken by the classic drop test. This means that the classic method suffers from the restricted number of measurements because of the destructive nature of the method and the restricted volume of the cryo-bath, the elevation of temperature during transferring the sample from the cryo-bath to the test machine, as well as the precision of the required energy. In the past, we have managed to use the permeability measurement as the indicator of DBTT, showing that the permeability method is better, faster, and cheaper than the classic drop test. The arrangement consisted of a primary and a secondary coil placed around the steel under test and immersed in a cryogenic bath. By cooling the steel in the cryo-bath or warming it up once removed, the permeability can be measured using a single sample (Figure 8). The correlation between drop tests and permeability is illustrated in Figure 9, demonstrating a good agreement between the classic and the magnetic method. The advantages of the magnetic permeability method include accurate measurement of the temperature and the ability to conduct a much higher number of measurements compared to the drop test. Apart from that, the magnetic method is faster, requiring not more than 30 min for the whole DBTT test, in comparison with the 4–6 h required for the drop test. Finally, it is cheaper for obvious reasons in terms of human hours required for the measurement, as well as in terms of the equipment required for the drop test in comparison with the M-H loop tests. The GMI sensor can effectively replace the primary and secondary coils, which greatly simplifies the magnetic method. In this setup, only a single sensor is used, located on top of the steel plate within a special packaging for this purpose. This simplifies the operator’s work: they only need to execute one single command to start the entire experiment, which lasts until the steel cools down or heats up. This process can be adopted in the quality management system of pipeline manufacturers, as well as other steel producers, in order to standardize the method, with the possibility of expanding the standardization to other areas such as hardness, microhardness, etc. The latter is also important: the magnetic field can be calibrated for hardness and microhardness in the same way as DBTT, compared to standard certified hardness and microhardness testing instruments. The microhardness measurement process can be realized by the GMI sensor due to the micron diameter of its internal magnetic core, which ranges from 5 µm to 15 µm.

**Figure 10:**
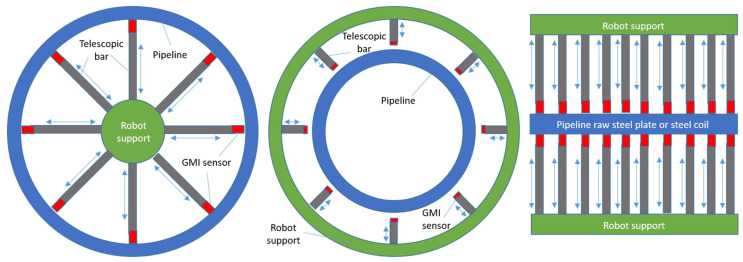
The robotic arms dedicated to the internal part of the pipeline (**left**), the external part of the pipeline (**middle**), and the incoming raw steel material (**right**).

The AMR sensor can be used in a completely different application because of its characteristics and cost. This can be a sensing element arranged in arrays to determine the non-uniformity of the three magnetic field components on top of the steel under test. In fact, this test is meaningful in two applications. The most important one is related to the quality inspection of the internal and external surface of the pipeline. For this purpose, telescopic robotic arms can be radially arranged to attach to the internal part of the pipeline using an internal tube/cylinder to support them at the middle of the pipeline. A similar robotic telescopic arm can be used to inspect the outside part of the pipeline by being fixed on an external ring that supports these arms. Bearing in mind the fact that the AMR sensor requires 1 mm to accurately determine the three magnetic field components and the fact that the internal coating of the pipelines is less than 0.2–0.3 mm, it is concluded that AMR sensors are suitable for use as the above-mentioned sensing elements. Apart from that, AMR sensors can also be used to inspect the quality of the incoming steel plate or steel coil raw material. For this reason, robotic telescopic bars in one or more lines are required to scan the surface of the steel under test. For all these applications, the required electronics provide signal processing, data transmission, and analysis without the need to actually excite the sensing elements, which are passive, as analyzed in the previous chapters. Figure 10 depicts these three applications of the AMR sensor in the pipeline manufacturing process.

When working with GMI sensors, it is better to utilize flexible electronics and self-produced glass-covered amorphous wires to ensure autonomy in production, maintenance, and RTD, as shown below. The cost of a glass-covered amorphous wire production line is in the order of EUR 100k for a production of 1 km per day or 200,000 sensing elements per day. The cost of a flexible electronics lab to support the manufacturing of 1000 sensors per day is in the order of EUR 300k. Thus, including annealing and property tailoring processes, as well as characterization tools like magnetization loops, magnetoimpedance loops, and calibration, the total capital expenses (CAPEX) for an entity are below EUR 1 M, allowing for a production of more than 200,000 sensors per year. In parallel, the needs for operational expenses (OPEX) are restricted to 4–5 engineers and 2–3 technicians working for the production of these sensors, allowing for a rough estimation between EUR 500k and EUR 1 M. Considering sales of at least 10,000 sensors per year at a price of EUR 1k each allows for a profit from the first year of operation.

When using AMR sensors, the devices should be purchased, as the cost of a production line can reach several billion euros. At the same time, the required quantity of AMR sensors per instrument to be developed is restricted to no more than 10,000–30,000 sensors per year. It is possible, or even preferable, to purchase AMR wafers as raw material and then proceed with the remaining AMR manufacturing steps, like cutting, wire bonding, packaging, and testing. The cost of such instrumentation is restricted to less than EUR 1 M, including the required clean or gray rooms and sensor calibration testers. In addition, the process of developing the robotic arms requires a CNC mechanical workshop, an electronics workshop, and maybe a hybrid or thick film or flexible electronics workshop, along with a computer lab for the preparation of the human–machine interface and required algorithms for the data analysis process, which is estimated to be another EUR 1 million. To these costs, the initial wafer cost has to be added, which is in the order of another EUR 1 M for 30,000 AMR sensors. Thus, the total CAPEX is estimated at EUR 3 M. For all this, a total of 4–5 engineers and 2–3 technicians is required, suggesting an operation cost (OPEX) in the order of EUR 0.5 M to EUR 1 M. Therefore, considering sales of such robotic systems, as 2–3 per year, at a price larger than EUR 1 M, a total amount of EUR 9 M per year is foreseen, thus allowing for a profit from the first year of operation.

However, from the above analysis, it is clear that GMI sensor production is a relatively low-risk and low-profit activity, while AMR sensor production is a high-risk, high-profit activity. Experimental work is in progress, and these results will be illustrated in future work.

## 5. Conclusions

The properties of the GMI and AMR sensors for the determination of magnetic properties are analyzed in this paper. The GMI sensor is a hybrid, macroscopic sensor with better sensitivity than the AMR sensor, which is an integrated microsensor. The GMI sensor exhibits high sensitivity, allowing the observation of interesting details in the residual stress profiles of the samples. However, its lower span is mainly suitable for laboratory use, while the AMR sensor can be used more widely in the field. Specific examples of their use for a pipeline manufacturer demonstrate the possibility of using the GMI sensor as a DBTT tester and the AMR sensor as the sensing core for robotic tests of the pipeline and the corresponding raw material.

## Figures and Tables

**Table 1:** Characteristics of the GMI and AMR sensors.

Properties	GMI Sensor	AMR Sensor
Sensitivity	10 pTHz^−1/2^ @ 1 Hz	50 nTHz^−1/2^ @ 1 Hz
Dynamic range	±0.1 mT	±10 mT
Uncertainty	~±1 nT	~+1 μT
Temperature coefficient	~500 ppm/K [16]	100 ppm/K

## Data Availability

Data are available at request by the author.
